# Oleoylethanolamide supplementation on cardiometabolic health: a systematic review and meta-analysis of randomized controlled trials

**DOI:** 10.3389/fnut.2025.1553288

**Published:** 2025-05-21

**Authors:** Hossein Bahari, Mostafa Shahraki Jazinaki, Ladan Aghakhani, Alireza Hatami, Iman Rahnama, Mahsa Malekahmadi

**Affiliations:** ^1^Transplant Research Center, Clinical Research Institute, Mashhad University of Medical Sciences, Mashhad, Iran; ^2^Student Research Committee, Mashhad University of Medical Sciences, Mashhad, Iran; ^3^Laparoscopy Research Center, Shiraz University of Medical Sciences, Shiraz, Iran; ^4^Imam Khomeini Hospital Complex, Tehran University of Medical Sciences, Tehran, Iran

**Keywords:** oleoylethanolamide, inflammation, oxidative stress, obesity, metabolic health, meta-analysis

## Abstract

**Background:**

Oleoylethanolamide (OEA) is a naturally occurring lipid that has been studied for its potential role in weight management and metabolic health. Through comprehensive meta-analysis, we aim to clarify the potential benefits of OEA in improving inflammation, oxidative stress, and metabolic parameters.

**Methods:**

To identify relevant randomized controlled trials (RCTs), a comprehensive search was conducted using Google Scholar and four databases: PubMed, Embase, Scopus, and Web of Science, up to November 2024. Eligible trials were detected by screening, and related data were extracted, respectively. Pooled effect sizes were calculated using meta-analyses and expressed as standard mean difference (SMD) with a 95% confidence interval (CI).

**Results:**

Ten trials (with 11 treatment arms) were eligible for inclusion in this review. Meta-analysis revealed that OEA supplementation led to a significant improvement in C-reactive protein (CRP), tumor necrosis factor-α (TNF-α), total antioxidant capacity (TAC), malondialdehyde (MDA), body weight, body mass index (BMI), waist circumference (WC), fat mass (FM), body fat percentage (BFP), triglycerides (TG), fasting blood glucose (FBG), insulin, and Homeostatic Model Assessment for Insulin Resistance (HOMA-IR) levels. However, no significant changes were observed in interleukin 6 (IL-6), fat-free mass (FFM), total cholesterol (TC), low-density lipoprotein cholesterol (LDL-C), high-density lipoprotein cholesterol (HDL-C), and hemoglobin A1C (HbA1c) following OEA intake.

**Conclusion:**

Supplementation with OEA may help improve glycemic control, weight loss, waist circumference, fat mass, fat percentage, inflammation, and oxidative stress. However, further research is needed to establish definitive conclusions regarding its efficacy and long-term benefits.

## 1 Introduction

Cardiometabolic health, as defined by the American Heart Association (AHA), encompasses the simultaneous presence of optimal levels of three clinical health factors: untreated blood pressure (<120/<80 mmHg), total cholesterol (TC) (<200 mg/dL), and fasting blood glucose (FBG) (<100 mg/dL), alongside four ideal health behaviors, namely a body mass index (BMI) below 25 kg/m^2^, non-smoking status, adequate physical activity, and adherence to dietary guidelines (1–3). Notably, parameters such as elevated glycemic indices, increased adiposity (e.g., weight, BMI, waist circumference, fat mass, and fat percentage), chronic inflammation, and oxidative stress are strongly associated with impaired cardiometabolic health and increased risk of cardiovascular disease (CVD) (4–8). Obesity and its related metabolic disturbances are among the most significant modifiable risk factors for cardiometabolic disease (9). Chronic low-grade inflammation and oxidative stress play central roles in the pathophysiology of these conditions. Consequently, targeting inflammation, insulin resistance, and body composition through lifestyle and dietary interventions has become a key strategy in managing cardiometabolic risk (10, 11).

Oleoylethanolamide (OEA), an endogenous fatty acid amide, is a bioactive mono-unsaturated lipid mediator that is part of the acylglycerol and N-acylethanolamine families, sharing structural similarities with endocannabinoids. OEA is produced from oleic acid and is synthesized in the gastrointestinal tract, fat tissues, neurons, and astrocytes. Oatmeal, nuts, and cocoa powder are dietary sources of OEA; however, their OEA content is low (less than 2 μg/g) (12–14). OEA has recently gained increased attention due to the extensive range of health benefits that have been well-documented in humans. Growing evidence has established the protective role of OEA in various areas, including inflammation and oxidative stress, triglyceride regulation, glycemic control, insulin resistance (IR), non-alcoholic fatty liver disease (NAFLD), weight loss, stimulation of lipolysis, and enhancement of fatty acid oxidation (12, 15–17).

OEA mainly functions by binding to the peroxisome proliferator-activated receptor alpha (PPAR-α), which is a nuclear receptor responsible for regulating lipid metabolism and maintaining energy balance. Activating PPAR-α receptors after exposure to OEA enhances the expression of genes related to fatty acid oxidation and lipolysis in white adipose tissue (12, 18). Furthermore, OEA acts as a ligand for PPAR-α, binding to its receptors and decreasing the production of pro-inflammatory cytokines and reactive oxygen species (ROS) (12).

Multiple studies have investigated the impact of OEA supplementation across different populations. For example, certain randomized controlled trials (RCTs) indicated a relationship between OEA levels and enhanced glycemic control, as well as lower blood glucose, insulin levels, and HOMA-IR in individuals with metabolic disorders (15, 17, 19). Additionally, in an RCT conducted by Laleh et al. (12), it was reported that supplementation with two 125 mg OEA capsules daily for 8 weeks in a group of 60 healthy obese individuals enhanced the expression of the PPAR-α gene and improved various anthropometric measurements, including weight, BMI, waist circumference, fat mass, and appetite sensations. In contrast, another study did not demonstrate any significant weight loss following the OEA intervention (19).

Furthermore, several earlier studies have found that OEA supplementation exhibits anti-inflammatory properties, leading to a significant decrease in serum levels of interleukin-6 (IL-6), tumor necrosis factor-alpha (TNF-α), and the expression of nuclear factor-kappa B (NF-κB) (12, 20). On the other hand, a study conducted by Tutunchi (10) found that treatment with OEA did not significantly affect inflammatory biomarkers, including high-sensitivity C-reactive protein (hs-CRP), IL-1β, IL-6, IL-10, and TNF-α.

Therefore, due to the inconsistency among studies, we conducted a comprehensive systematic review and meta-analysis of published randomized controlled trials (RCTs) to offer clearer insights into the effects of OEA supplementation on cardiometabolic health.

## 2 Materials and methods

### 2.1 Protocol and registration

We design and carry out all steps of our systematic review based on the Preferred Reporting Items of Systematic Reviews and Meta-Analysis (PRISMA) guidelines (21). Moreover, we registered the protocol for conducting this review in PROSPERO databases with the following registration ID: CRD42024622528.

### 2.2 Search strategy and study identification

We comprehensively searched Embase, PubMed, Scopus, and Web of Science ISI databases to find the RCTs investigating the impact of oleoylethanolamide (OEA) supplementation on cardiometabolic parameters in adults until November 2024. This search had no time or language restrictions. Two authors (M.Sh.J and H.B) independently screened the obtained papers based on the eligibility criteria by using the EndNote software. The implemented search strategy in each database consisted of related keywords, including (“oleoylethanolamide” OR “OEA” OR “N-oleyl-phosphatidylethanolamine” OR “NOPE”) AND (“intervention” OR “clinical trial” OR “controlled trial” OR “RCT” OR “randomized controlled trial” OR “randomized” OR “parallel” OR “cross-over”). We checked the reference lists of all included trials to avoid missing eligible RCTs. In addition, the Google Scholar search engine was manually searched.

### 2.3 Eligibility criteria

The inclusion criteria of this review were designed based on the PICOS framework as follows (22): (a) P (population): Adults (aged >18 years), I (intervention): oleoylethanolamide supplementation, C (comparison): control groups, O (outcomes): cardiometabolic markers, and S (study type): RCTs with at least 1 week of intervention duration. Performing co-supplementation, trials with less than 1 week duration, intervention conducted on non-adults, and RCTs that did not report the changes in cardiometabolic markers as mean ± SD (or SE, or IQR) were excluded from this meta-analysis. Interventions on animals, review articles, and observational studies (such as case–control, cross-sectional, and cohort) were other exclusion criteria for this review.

### 2.4 Data extraction

Two authors independently extracted the related data from the included studies, including the country of study, publication year, name of the first author, sample size, number of individuals in each group, characteristics of the population (health status, mean body mass index (BMI), and mean age), intervention features of OEA (duration and dosage), type of control group, main outcomes, and mean change and SD of related markers during the intervention (or measures in baseline and the end of intervention). Disagreements between the two authors in data extraction were discussed until a consensus was reached.

### 2.5 Risk of bias assessment

The Cochrane risk-of-bias tool for randomized trials (RoB 2) was our approach for assessing the risks of bias in included studies (23). This framework evaluated the risk of bias in five domains, including bias due to missing outcome data, bias in the selection of the reported result, bias arising from the randomization process, bias in the measurement of the outcome, and bias due to deviations from intended interventions. Based on the risk of bias assessment in each domain, the general risk of bias was determined for each included study as high, with some concerns, or low.

### 2.6 Statistical analysis and GRADE scoring

Statistical analysis in this meta-analysis was conducted using STATA, version 17 (Stata Corp, College Station, TX, USA). In all analyses, *p*-values <0.05 are considered statistically significant. In this meta-analysis, the pooled effect sizes were expressed as standard mean difference (SMDs) and 95% confidence interval (CI). The mean and SD of changes for cardioembolic outcomes in each group were used to calculate pooled effect sizes by applying the random effects model (23). If the mean and SDs of each outcome were not reported directly, we estimated them by the following formula: (Mean change: final value-baseline value, SD: square root [(SDbaseline)^2^ + (SDfinal)^2^ − (2 × R × SDbaseline × SDfinal)]) (24). Reported 95% CIs, standard errors (SEs), or interquartile ranges (IQRs) were converted to SDs based on the method proposed by Hozo et al. (25). The heterogeneity among the included studies was assessed by performing Cochran’s Q-test and interpretation of *I*^2^ statistic (*I*^2^ > 50% and *p* < 0.05 indicated a significant heterogeneity) (26). To detect the possible source of heterogeneity, subgroup analyses were performed based on predefined criteria, including gender (both male and female), OEA dosage (<250 and ≥250 mg/day), intervention duration (<8 and ≥8 weeks), and baseline BMI (obesity, overweight, and normal). The publication bias in each outcome was assessed by performing the Egger test and visual inspection of the related funnel plot (27). The impact of one specific effect size on our findings was evaluated by conducting the sensitivity analysis. We applied the GRADE (Grading of Recommendations Assessment, Development, and Evaluation) protocol to assess the quality of evidence on the impact of OEA on cardiometabolic outcomes (28). This framework evaluated the limitations of the trial quality in five sections, including inconsistency, publication bias, indirectness, and imprecision. The overall quality of evidence for each outcome was classified into four levels: high, moderate, low, and very low.

## 3 Results

### 3.1 Study selection

**Figure 1:**
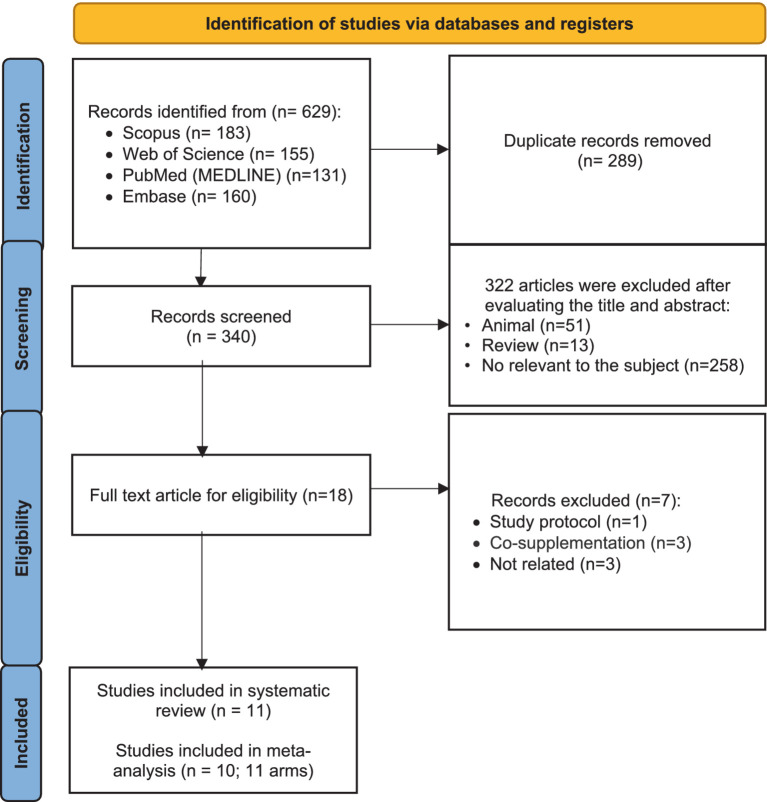
Flow chart of study selection for inclusion trials in the systematic review.

From 629 studies that were found from searches in databases, 289 duplicated articles were removed. Then, 340 remaining studies were screened based on their titles and abstracts. In this step, 322 were excluded for not meeting the inclusion criteria. The full text of the 18 remaining articles was read, which led to the exclusion of seven papers due to being study protocol articles (*n* = 1), conducting co-supplementation (*n* = 3), and no reporting required data (*n* = 3). Eleven trials were included in the systematic review. Finally, 10 studies (with 11 effect sizes) were included in the meta-analysis (Figure 1) (12, 15–17, 19, 20, 29–32).

### 3.2 Study characteristics

Eligible trials were published between 2018 (12, 31) and 2024 (16, 19). All included studies were conducted in Iran and had a parallel design. Among the included trials, three had a triple-blind design (15, 20, 32), and others were double-blinded. Moreover, two studies were performed only on women (19, 30), while the rest were conducted on both genders. In the included studies, intervention with OEA was performed in healthy obese people (12, 16, 31), participants with acute ischemic stroke (33), obesity and NAFLD (15, 20, 32), COVID-19 (29), prediabetes (17), primary dysmenorrhea (30), and Polycystic ovary syndrome (PCOS) (19). The sample sizes of the included effect sizes were between 20 (29) and 87 participants (19). The mean age of participants varied from 20.6 (30) to 69.15 years (33). Moreover, the mean BMI of individuals in eligible trials was between 23.2 (30) and 34.9 kg/m^2^ (12). The duration of receiving OEA ranged from 3 days (33) to 12 weeks (15, 20, 32), and the OEA dosage was between 125 (17, 19, 30) and 600 mg/day (33). Table 1 provides a summary of the included trial features.

**Table 1:** Characteristic of included studies in the systematic review.

Studies	Country	Study design	Participant	Sex	Sample size		Trial duration (Week)	Means age		Means BMI		Intervention			Measured outcomes
					IG	CG		IG	CG	IG	CG	Type	Dose (mg/day)	Control group	
Laleh et al. 2018 (12)	Iran	Parallel, R, PC, DB	Healthy obese people	M/F (F: 34, M: 22)	27	29	8	37.4	38.1	34.7 ± 2.4	35.1 ± 2.8	OEA	250	Placebo (starch)	Body weight, BMI, WC, FM, BFP, FFM
Payahoo et al. 2018 (31)	Iran	Parallel, R, PC, DB	Healthy obese people	M/F (F: 34, M: 22)	27	29	8	37.3	38.1	34.6 ± 2.4	35.1 ± 2.8	OEA	250	Placebo (starch)	MDA, TAC, CRP, TNF-a, IL-6
Tutunchi et al. 2020 (15)	Iran	Parallel, R, PC, TB	Obese and NAFLD	M/F (F: 37, M: 39)	38	38	12	40.8	42.1	33.1 ± 3.2	33.4 ± 3.2	OEA	250	Placebo (starch)	TG, Total cholesterol, LDL, HDL, FBG, Insulin, HbA1c, HOMA-IR, body weight, BMI, WC
Tutunchi et al. 2021 (20)	Iran	Parallel, R, PC, TB	Obese and NAFLD	M/F (F: 37, M: 39)	38	38	12	40.8	42.1	33.1 ± 3.2	33.4 ± 3.2	OEA	250	Placebo (starch)	FM, BFP, FFM
Akbari et al. 2022 (29)	Iran	Parallel, R, PC, DB	COVID-19	M/F (F: 20, M: 10)	10	10	2	38.9	44.4	23.8 ± 3.7	25.2 ± 2.6	OEA	400	routine treatments	CRP, IL-6, BMI
Akbari et al. 2022 (29)	Iran	Parallel, R, PC, DB	COVID-19	M/F (F: 20, M: 10)	20	10	2	43.8	44.1	24.6 ± 3.9	24.4 ± 3.7	Boron + OEA	500	Boron	CRP, IL-6, BMI
Sabahi et al. 2022 (33)	Iran	Parallel, R, PC, DB	Acute Ischemic Stroke	M/F (F: 19, M: 21)	20	20	3 days	67.2	68.6	26.4 ± 4.3	27.2 ± 3.7	OEA	300	Placebo	TG, Total cholesterol, HDL, CRP, IL-6, MDA, TAC
Sabahi et al. 2022 (33)	Iran	Parallel, R, PC, DB	Acute Ischemic Stroke	M/F (F: 19, M: 21)	20	20	3 days	69.7	68.6	27.1 ± 3.4	27.2 ± 3.7	OEA	600	Placebo	TG, Total cholesterol, HDL, CRP, IL-6, MDA, TAC
Pouryousefi et al. 2022 (17)	Iran	Parallel, R, PC, DB	Prediabetes	M/F (F: 22, M: 21)	22	21	8	49.6	49.7	27.4 ± 1.0	27.2 ± 1.9	OEA	125	Placebo (wheat flour)	Body weight, BMI, FBG, Insulin, HbA1c, HOMA-IR, CRP
Kazemi et al. 2022 (30)	Iran	Parallel, R, PC, DB	Primary dysmenorrhea	F (F: 43)	22	21	8	20.8	20.5	23.3 ± 1.1	23.1 ± 1.4	OEA	125	Placebo (wheat flour)	MDA, TAC, CRP, TNF-a,
Tutunchi et al. 2023 (32)	Iran	Parallel, R, PC, TB	Obese and NAFLD	M/F (F: 32, M: 28)	30	30	12	41.7	42.3	33.7 ± 6.6	35.5 ± 7.1	OEA	250	Placebo (starch)	CRP, IL-6, TNF-a, MDA, TAC
Ostadrahimi et al. 2024 (16)	Iran	Parallel, R, PC, DB	Healthy obese people	M/F (F: 34, M: 22)	27	29	8	37.3	38.1	34.6 ± 2.4	35.1 ± 2.8	OEA	250	Placebo (starch)	TG, Total cholesterol, LDL, HDL, FBG
Shivyari et al. 2024 (19)	Iran	Parallel, R, PC, DB	PCOS	F (F: 87)	44	43	8	27.3	29.1	28 ± 0.3	28.1 ± 0.2	OEA	125	Placebo (wheat flour)	FBG, Insulin, HOMA-IR, CRP, TNF-a, body weight, BMI, MDA, TAC

IG, intervention group; CG, control group; TB, triple-blinded; DB, double-blinded; PC, placebo-controlled; CO, controlled; R, randomized; BMI, body mass index; WC, waist circumference; FM, fat mass; BFP, body fat percentage; FFM, fat-free mass; TG, triglycerides; LDL, low-density lipoprotein; HDL, high-density lipoprotein; FBG, fasting blood glucose; HbA1c, hemoglobin A1c; HOMA-IR, Homeostatic Model Assessment for Insulin Resistance; CRP, c-reactive protein; IL-6, interleukin-6; TNF-a, tumor necrosis factor-a; MDA, malondialdehyde; TAC, total antioxidant capacity.

The risk of bias assessment, conducted using the RoB 2 approach, revealed that the overall risk of bias was high for two of the included trials (29, 30) and raised some concerns for another two (20, 31). However, the remaining eligible trials were identified as having a low risk of bias. Details of the risk of bias assessment are presented in Table 2.

**Table 2:** Risk of bias assessment.

Study	Bias arising from the randomization process	Bias in selection of the reported result	Bias due to deviations from intended interventions	Bias in measurement of the outcome	Bias due to missing outcome data	Overall risk of bias
Laleh et al. 2018 (12)	L	L	L	U	L	Low
Payahoo et al. 2018 (31)	U	L	L	U	L	Some concerns
Tutunchi et al. 2020 (15)	L	L	L	L	L	Low
Tutunchi et al. 2021 (20)	L	H	L	L	L	Some concerns
Akbari et al. 2022 (29)	L	H	L	U	L	High
Sabahi et al. 2022 (33)	L	L	L	U	L	Low
Pouryousefi et al. 2022 (17)	L	L	L	U	L	Low
Kazemi et al. 2022 (30)	L	H	L	U	L	High
Tutunchi et al. 2023 (32)	L	L	L	L	L	Low
Ostadrahimi et al. 2024 (16)	L	L	L	U	L	Low
Shivyari et al. 2024 (19)	L	L	L	U	L	Low

L; low risk of bias; H, high risk of bias; U, unclear risk of bias.

Overall Low Risk < 2 unclear risk of bias and no high risk of bias.

Overall Some concerns = 2 unclear risk of bias and no high risk of bias.

Overall High Risk > 2 unclear risk of bias or more than one high risk of bias.

### 3.3 Meta-analysis

#### 3.3.1 Impact of OEA supplementation on inflammatory and oxidative stress markers

##### 3.3.1.1 Effect of OEA supplementation on CRP levels

**Figure 2:**
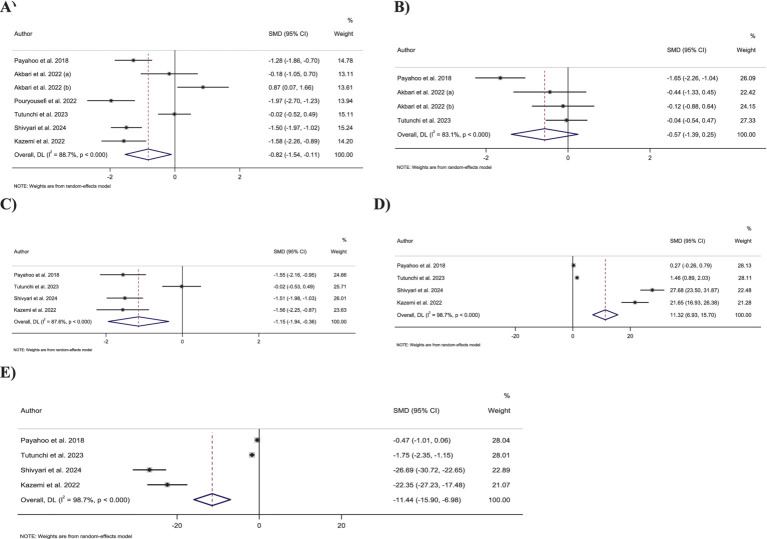
Forest plot detailing standardized mean difference and 95% confidence intervals (CIs) for the effect of oleoylethanolamide intake on **(A)** CRP; **(B)** IL-6; **(C)** TNF-α; **(D)** TAC; **(E)** MDA.

Meta-analyzing seven effect sizes showed that OEA intake led to a significant decrease in CRP levels compared to control groups (SMD: −0.82; 95% CI: −1.53 to −0.11; *p* = 0.02) (Figure 2A). However, significant heterogeneity was observed between the included effect sizes (*I*^2^ = 88.7%, *p* < 0.001). Subgroup analyses demonstrated that OEA consumption had no significant effect on CRP levels in trials conducted on both genders, participants with normal weight and obesity, trials with less than 8 weeks duration, or with ≥250 mg/day dosage of OEA (Table 3).

**Table 3:** Subgroup analyses of oleoylethanolamide on cardiometabolic health.

	Number of effect sizes	SMD (95%CI)	*p*-value	Heterogeneity		
				*p* heterogeneity	*I*^2^	*p* between subgroups
Oleoylethanolamide intake on CRP						
Overall effect	7	−0.82 (−1.53, −0.11)	0.024	<0.001	88.7%	
Sex						
Both	5	−0.52 (−1.45, 0.40)	0.267	<0.001	89.4%	0.051
Female	2	−1.52 (−1.91, −1.13)	<0.001	0.85	0.0%	
Trial duration (week)						
≥8	5	−1.24 (−1.93, −0.55)	<0.001	<0.001	85.6%	0.01
<8	2	0.36 (−0.65, 1.38)	0.485	0.08	66.4%	
Intervention dose (mg/day)						
≥250	4	−0.17 (−1.04, 0.69)	0.696	<0.001	85.5%	0.003
<250	3	−1.62 (−1.96, −1.27)	<0.001	0.56	0.0%	
Baseline BMI (kg/m^2^)						
Normal (18.5–24.9)	3	−0.30 (−1.78, 1.16)	0.684	<0.001	90.6%	0.09
Overweight (25–29.9)	2	−1.64 (−2.07, −1.21)	<0.001	0.29	10.1%	
Obese (>30)	2	−0.64 (−1.87, 0.59)	0.311	0.001	90.4%	
Oleoylethanolamide intake on IL-6						
Overall effect	4	−0.57 (−1.38, 0.24)	0.17	<0.001	83.1%	
Trial duration (week)						
≥8	2	−0.83 (−2.41, 0.74)	0.30	<0.001	93.7%	0.50
<8	2	−0.25 (−0.83, 0.32)	0.38	0.59	0.0%	
Baseline BMI (kg/m^2^)						
Normal (18.5–24.9)	2	−0.25 (−0.83, 0.32)	0.38	0.59	0.0%	0.50
Obese (>30)	2	−0.83 (−2.41, 0.74)	0.30	<0.001	93.7%	
Oleoylethanolamide intake on TNF-α						
Overall effect	4	−1.15 (−1.94, −0.35)	0.005	<0.001	87.6%	
Sex						
Both	2	−0.77 (−2.28, 0.72)	0.31	<0.001	93.2%	0.34
Female	2	−1.52 (−1.91, −1.13)	<0.001	0.89	0.0%	
Intervention dose (mg/day)						
≥250	2	−0.77 (−2.28, 0.72)	0.31	<0.001	93.2%	0.34
<250	2	−1.52 (−1.91, −1.13)	<0.001	0.89	0.0%	
Oleoylethanolamide intake on body weight						
Overall effect	4	−0.26 (−0.51, −0.02)	0.033	0.41	0.0%	
Intervention dose (mg/day)						
≥250	2	−0.42 (−0.80, −0.03)	0.033	0.26	19.1%	0.23
<250	2	−0.10 (−0.45, 0.23)	0.536	0.85	0.0%	
Baseline BMI (kg/m^2^)						
Overweight (25–29.9)	2	−0.10 (−0.45, 0.23)	0.536	0.85	0.0%	0.23
Obese (>30)	2	−0.42 (−0.80, −0.03)	0.033	0.26	19.1%	
Oleoylethanolamide intake on BMI						
Overall effect	6	−0.48 (−0.82, −0.14)	0.005	0.07	51.1%	
Trial duration (week)						
≥8	4	−0.56 (−0.98, −0.14)	0.008	0.04	63.5%	0.29
<8	2	−0.18 (−0.76, 0.38)	0.525	0.63	0.0%	
Intervention dose (mg/day)						
≥250	4	−0.48 (−0.78, −0.18)	0.002	0.40	0.0%	0.94
<250	2	−0.52 (−1.49, 0.45)	0.296	0.009	85.4%	
Baseline BMI (kg/m^2^)						
Normal (18.5–24.9)	2	−0.18 (−0.76, 0.38)	0.525	0.63	0.0%	0.53
Overweight (25–29.9)	2	−0.52 (−1.49, 0.45)	0.296	0.009	85.4%	
Obese (>30)	2	−0.58 (−0.98, −0.18)	0.004	0.25	23.6%	
Oleoylethanolamide intake on waist circumference						
Overall effect	2	−0.91 (−1.39, −0.42)	<0.001	0.18	44.3%	
Oleoylethanolamide intake on fat mass						
Overall effect	2	−0.53 (−0.87, −0.17)	0.003	0.63	0.0%	
Oleoylethanolamide intake on body fat percentage						
Overall effect	2	−0.46 (−0.80, −0.11)	0.009	0.35	0.0%	
Oleoylethanolamide intake on fat-free mass						
Overall effect	2	0.01 (−0.32, 0.35)	0.931	0.35	0.0%	
Oleoylethanolamide intake on TAC						
Overall effect	4	11.31 (6.92, 15.70)	<0.001	<0.001	98.7%	
Sex						
Both	2	0.86 (−0.31, 2.03)	0.151	0.003	89.0%	0.001
Female	2	24.77 (18.86, 30.67)	<0.001	0.061	71.5%	
Intervention dose (mg/day)						
≥250	2	0.86 (−0.31, 2.03)	0.151	0.003	89.0%	0.001
<250	2	24.77 (18.86, 30.67)	<0.001	0.061	71.5%	
Oleoylethanolamide intake on MDA						
Overall effect	4	−11.44 (−15.90, −6.97)	<0.001	<0.001	98.7%	
Sex						
Both	2	−1.10 (−2.35, 0.14)	0.083	0.002	89.7%	0.001
Female	2	−24.74 (−28.96, −20.52)	<0.001	0.18	44.4%	
Intervention dose (mg/day)						
≥250	2	−1.10 (−2.35, 0.14)	0.083	0.002	89.7%	0.001
<250	2	−24.74 (−28.96, −20.52)	<0.001	0.18	44.4%	
Oleoylethanolamide intake on triglycerides						
Overall effect	2	−0.45 (−0.79, −0.10)	0.011	0.83	0.0%	
Oleoylethanolamide intake on total cholesterol						
Overall effect	2	0.12 (−0.21, 0.46)	0.477	0.50	0.0%	
Oleoylethanolamide intake on LDL						
Overall effect	2	0.25 (−0.27, 0.78)	0.352	0.128	56.8%	
Oleoylethanolamide intake on HDL						
Overall effect	2	0.51 (−0.94, 1.95)	0.491	<0.001	93.8%	
Oleoylethanolamide intake on FBG						
Overall effect	4	−1.54 (−2.55, −0.52)	0.003	<0.001	92.2%	
Intervention dose (mg/day)						
≥250	2	−0.68 (−1.03, −0.33)	<0.001	0.46	0.0%	0.24
<250	2	−2.66 (−5.93, 0.61)	0.112	<0.001	96.6%	
Baseline BMI (kg/m^2^)						
Overweight (25–29.9)	2	−2.66 (−5.93, 0.61)	0.112	<0.001	96.6%	0.24
Obese (>30)	2	−0.68 (−1.03, −0.33)	<0.001	0.46	0.0%	
Oleoylethanolamide intake on insulin						
Overall effect	3	−2.03 (−3.40, −0.66)	0.004	<0.001	93.4%	
Oleoylethanolamide intake on HbA1c						
Overall effect	2	−1.22 (−3.34, 0.88)	0.255	<0.001	95.4%	
Oleoylethanolamide intake on HOMA-IR						
Overall effect	3	−5.46 (−10.07, −0.85)	0.020	<0.001	98.8%	

WMD, weighted mean differences; CI, confidence interval; BMI, body mass index; LDL, low-density lipoprotein; HDL, high-density lipoprotein; FBG, fasting blood glucose; HbA1c, hemoglobin A1c; HOMA-IR, Homeostatic Model Assessment for Insulin Resistance; CRP, c-reactive protein; IL-6, interleukin-6; TNF-a, tumor necrosis factor-a; MDA, malondialdehyde; TAC, total antioxidant capacity.

##### 3.3.1.2 Effect of OEA supplementation on IL-6 levels

Combining four effect sizes demonstrated that OEA intake had no significant impact on IL-6 levels compared to control groups (SMD: −0.57; 95% CI: −1.38 to 0.24; *p* = 0.17) (Figure 2B). Moreover, there was significant heterogeneity among the included effect sizes (*I*^2^ = 83.1%, *p* < 0.001). Furthermore, no significant changes in IL-6 levels were reported in any of the predefined subgroups (Table 3).

##### 3.3.1.3 Effect of OEA supplementation on TNF-α levels

Pooling four effect sizes reported a significant reduction in the TNF-α levels in groups that received OEA compared to the control groups (SMD: −1.15; 95% CI: −1.94 to −0.35; *p* = 0.005) (Figure 2C). Meanwhile, a significant heterogeneity was detected between included effect sizes (*I*^2^ = 87.6%, *p* < 0.001). However, subgroup analysis demonstrated that OEA consumption in trials conducted on both sexes or with an OEA dosage of ≥250 mg/day had no significant influence on TNF-α levels (Table 3).

##### 3.3.1.4 Effect of OEA supplementation on TAC levels

Combining four effect sizes revealed that OEA intake led to a significant increase in TAC levels compared to the control groups (SMD: 11.31; 95% CI: 6.92–15.70; *p* < 0.001) (Figure 2D). However, significant heterogeneity existed between the included effect sizes (*I*^2^ = 98.7%, *p* < 0.001). Subgroup analyses showed that OEA consumption had no significant impact on TAC levels in trials conducted in both genders or with intervention OEA dosage of ≥250 mg/day (Table 3).

##### 3.3.1.5 Effect of OEA supplementation on MDA levels

Meta-analyzing four effect sizes demonstrated that OEA intake had a significant reduction in impact on MDA levels in comparison to the control groups (SMD: −11.44; 95% CI: −15.90 to −6.97; *p* < 0.001) (Figure 2E). Meanwhile, significant heterogeneity was observed among the included effect sizes (*I*^2^ = 98.7%, *p* < 0.001). However, OEA consumption did not significantly impact MDA levels in trials conducted on both genders or studies with a dosage of ≥250 mg/day OEA (Table 3).

#### 3.3.2 Impact of OEA supplementation on anthropometric indices and body composition

##### 3.3.2.1 Effect of OEA supplementation on BMI

**Figure 3:**
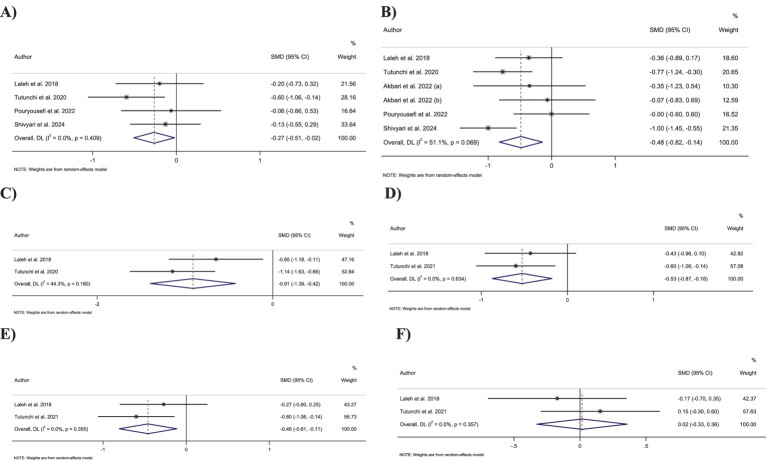
Forest plot detailing standardized mean difference and 95% confidence intervals (CIs) for the effect of oleoylethanolamide intake on **(A)** body weight; **(B)** BMI; **(C)** waist circumference; **(D)** fat mass; **(E)** body fat percentage; **(F)** fat-free mass.

Combining six effect sizes demonstrated that OEA intake had a significant reduction effect on BMI compared to the control groups (SMD: −0.48; 95% CI: −0.82 to −0.14; *p* = 0.005) (Figure 3A). Moreover, no significant heterogeneity was detected between the included effect sizes (*I*^2^ = 51.1%, *p* = 0.07). However, based on the subgroup analyses, OEA consumption had no significant impact on the BMI in the trials conducted on individuals with normal BMI and overweight, with a duration of <8 weeks or with OEA dosage of <250 mg/day (Table 3).

##### 3.3.2.2 Effect of OEA supplementation on body weight

Pooling four effect sizes revealed that OEA intake led to a significant decrease in body weight in comparison to the control groups (SMD: −0.26; 95% CI: −0.51 to −0.02; *p* = 0.03) (Figure 3B). Moreover, no significant heterogeneity was detected between the included effect sizes (*I*^2^ = 0.0%, *p* = 0.41). Subgroup analyses showed a non-significant impact of OEA consumption on body weight in trials conducted on participants who were overweight or with an OEA dosage of <250 mg/day.

##### 3.3.2.3 Effect of OEA supplementation on WC

Merging two effect sizes showed that OEA intake led to a significant decrease in WC compared to the control groups (SMD: −0.91; 95% CI: −1.39 to −0.42; *p* < 0.001) (Figure 3C). Furthermore, no significant heterogeneity was detected between pooled effect sizes (*I*^2^ = 44.3%, *p* = 0.18).

##### 3.3.2.4 Effect of OEA supplementation on FM

Combining two effect sizes demonstrated that OEA intake had a significant reduction effect on FM (SMD: −0.53; 95% CI: −0.87 to −0.17; *p* = 0.003) (Figure 3D). Furthermore, the heterogeneity among included effect sizes was not significant (*I*^2^ = 0.0%, *p* = 0.63).

##### 3.3.2.5 Effect of OEA supplementation on fat percentage

Merging two effect sizes revealed that OEA intake led to a significant decrease in fat percentage compared to the control groups (SMD: −0.46; 95% CI: −0.80 to −0.11; *p* = 0.009) (Figure 3E). Moreover, there was no significant heterogeneity between included effect sizes (*I*^2^ = 0.0%, *p* = 0.35).

##### 3.3.2.6 Effect of OEA supplementation on fat-free mass

Meta-analyzing the two effect sizes mentioned no significant changes in fat-free mass in groups receiving the OEA compared to control groups (SMD: 0.01; 95% CI: −0.32 to 0.35; *p* = 0.93) (Figure 3F). Furthermore, no significant heterogeneity was detected among the included effect sizes (*I*^2^ = 0.0%, *p* = 0.35).

#### 3.3.3 Impact of OEA supplementation on glycemic control markers

##### 3.3.3.1 Effect of OEA supplementation on FBG levels

**Figure 4:**
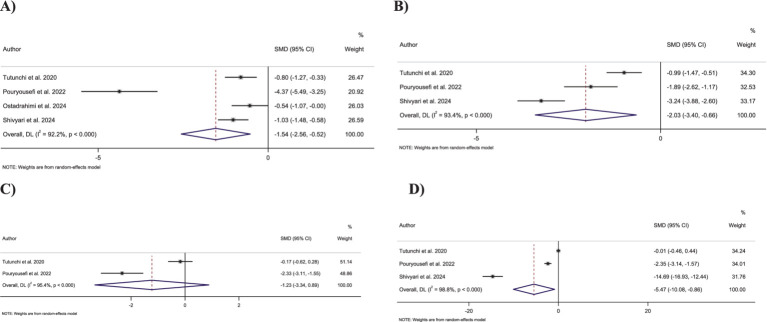
Forest plot detailing standardized mean difference and 95% confidence intervals (CIs) for the effect of oleoylethanolamide intake on **(A)** FBG; **(B)** Insulin; **(C)** HbA1c; **(D)** HOMA-IR.

Pooling four effect sizes revealed that OEA intake led to a significant decrease in FBG levels compared to the control group (SMD: −1.54; 95% CI: −2.55 to −0.52; *p* = 0.003) (Figure 4A). However, a significant heterogeneity was observed between pooled effect sizes (*I*^2^ = 92.2%, *p* < 0.001).

##### 3.3.3.2 Effect of OEA supplementation on insulin levels

Combining three effect sizes showed that OEA intake significantly decreased insulin levels compared to the control groups (SMD: −2.03; 95% CI: −3.40 to −0.66; *p* = 0.004) (Figure 4B). However, significant heterogeneity was detected between the merged effect sizes (*I*^2^ = 93.4%, *p* < 0.001).

##### 3.3.3.3 Effect of OEA supplementation on HbA1c levels

Merging two effect sizes showed no significant changes in HbA1c levels followed by OEA intake compared to the control group (SMD: −1.22; 95% CI: −3.34 to 0.88; *p* = 0.25) (Figure 4C). Furthermore, there was a significant heterogeneity among the combined effect sizes (*I*^2^ = 95.4%, *p* < 0.001).

##### 3.3.3.4 Effect of OEA supplementation on HOMA-IR

Combining three effect sizes demonstrated a significant reduction in the impact of OEA intake on HOMA-IR compared to the control groups (SMD: −5.46; 95% CI: −10.07 to −0.85; *p* = 0.02) (Figure 4D). However, a significant heterogeneity was reported between merged effect sizes (*I*^2^ = 98.8%, *p* < 0.001).

#### 3.3.4 Impact of OEA supplementation on lipid profile

##### 3.3.4.1 Effect of OEA supplementation on TC levels

**Figure 5:**
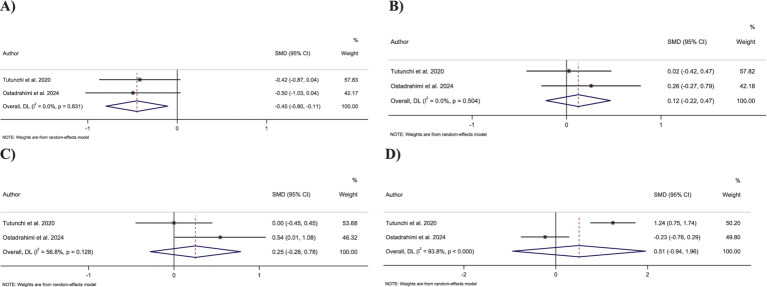
Forest plot detailing standardized mean difference and 95% confidence intervals (CIs) for the effect of oleoylethanolamide intake on **(A)** triglycerides; **(B)** total cholesterol; **(C)** LDL-C; and **(D)** HDL-C.

Merging two effect sizes showed that OEA intake had no significant impact on TC levels (SMD: 0.12; 95% CI: −0.21 to 0.46; *p* = 0.47) (Figure 5A). Furthermore, no significant heterogeneity was detected between the included effect sizes (*I*^2^ = 0.0%, *p* = 0.50).

##### 3.3.4.2 Effect of OEA supplementation on TG levels

Combining two effect sizes demonstrated that OEA intake led to a significant decrease in TG levels compared to the control groups (SMD: −0.45; 95% CI: −0.79 to −0.10; *p* = 0.01) (Figure 5B). Moreover, no significant heterogeneity was observed among pooled effect sizes (*I*^2^ = 0.0%, *p* = 0.83).

##### 3.3.4.3 Effect of OEA supplementation on LDL-C levels

Pooling two effect sizes showed no significant changes in LDL-C levels followed by OEA intake compared to control groups (SMD: 0.25; 95% CI: −0.27 to 0.78; *p* = 0.35) (Figure 5C). Moreover, no significant heterogeneity exists between the included effect sizes (*I*^2^ = 56.8%, *p* = 0.12).

##### 3.3.4.4 Effect of OEA supplementation on HDL-C levels

Combining two effect sizes revealed that OEA intake had no significant impact on HDL-C levels compared to the control groups (SMD: 0.51; 95% CI: −0.94 to 1.95; *p* = 0.49) (Figure 5D). Furthermore, a significant heterogeneity was detected among pooled effect sizes (*I*^2^ = 93.8%, *p* < 0.001).

### 3.4 Sensitivity analysis

To assess the influence of each effect size on the overall effect size for each outcome, a sensitivity analysis was carried out. Sensitivity analysis showed that the impact of OEA consumption on CRP levels changes significantly after excluding the studies by Payahoo et al. (SMD: −0.74; 95% CI: −1.58 to 0.10) (31), Pouryousefi et al. (SMD: −0.70; 95% CI: −1.52 to 0.12) (17), or Kazemi et al. (SMD: −0.69; 95% CI: −1.49 to 0.10) (30). Moreover, after removing the studies by Payahoo et al. (SMD: −1.01; 95% CI: −2.05 to 0.01) (31) or Shivyari et al. (SMD: −1.02; 95% CI: −2.10 to 0.04) (19), the overall effect size of OEA intake on TNF-α levels significantly changed. In addition, the overall effect sizes for TAC and MDA were significantly changed after excluding the studies by Tutunchi et al. (SMD: 16.44; 95% CI: −3.47 to 36.37, SMD: −16.41; 95% CI: −36.01 to 3.18, respectively) (32), or Payahoo et al. (SMD: 16.84; 95% CI: −2.12 to 35.80, SMD: −16.83; 95% CI: −35.41 to 1.73 (31), respectively).

Furthermore, the pooled effect size of OEA consumption on body weight after excluding Laleh et al. (SMD: −0.28; 95% CI: −0.61 to 0.05) (12) or Tutunchi et al. (SMD: −0.13; 95% CI: −0.42 to 0.15) (15) changed significantly. Moreover, removing Tutunchi et al., the overall effect size for fat percentage (SMD: −0.40; 95% CI: −0.81 to 0.01), FM (SMD: −0.42; 95% CI: −0.96 to 0.10), or fat percentage (SMD: −0.27; 95% CI: −0.79 to 0.25) significantly changed (15). Sensitivity analysis reported significant changes in overall effect size for insulin levels after removing the study by Pouryousefi et al. (SMD: −2.10; 95% CI: −4.31 to 0.10) (17). Moreover, a significant change was mentioned in the pooled effect size of HbA1c, followed by excluding the study by Tutunchi et al. (SMD: −2.33; 95% CI: −3.11 to −1.54). Furthermore, this analysis demonstrated that removing the studies by Tutunchi et al. (SMD: −8.47; 95% CI: −20.56 to 3.61), Pouryousefi et al. (SMD: −7.30; 95% CI: −21.68 to 7.07), or Shivyari et al. (SMD: −1.15; 95% CI: −3.45 to 1.13) (19) led to a significant change in HOMA-IR.

Excluding the study by Ostadrahimi et al. led to significant changes in the overall effect sizes for TG (SMD: −0.41; 95% CI: −0.87 to 0.03) and HDL (SMD: 1.24; 95% CI: 0.75–1.73) (16). In addition, the pooled effect size for TG significantly changes after removing Tutunchi et al. (15) (SMD: -0.49; 95% CI: −1.02 to 0.03).

However, the overall effect size for other outcomes was not significantly influenced by the quality of certain trials.

### 3.5 Publication bias

Assessing publication bias by Egger’s test revealed a significant publication bias in effect sizes that pooled for estimation of the impact of OEA intake on TAC (P_Egger_ = 0.02), MDA (P_Egger_ = 0.02) and HOMA-IR (P_Egger_ = 0.03). However, no significant publication bias was observed in evidence for the impact of OEA consumption on other outcomes (Supplementary Figure 1).

### 3.6 GRADE analysis

Evidence quality assessment based on the GRADE protocol identified the quality of evidence for TG, FM, and FMP as high. Moreover, the evidence for TC, BMI, WC, and FFM is considered moderate quality. Furthermore, evidence quality for LDL-C, insulin, CRP, and TNF-a were classified as low, and for HDL-C, HbA1c, HOMA-IR, IL-6, TAC, and MDA as very low quality (GRADE profile are provided in Supplementary Table 1).

## 4 Discussion

OEA reduced the levels of inflammatory markers, including CRP and TNF-α, in the intervention group compared to the control group. The reduction in CRP and TNF-α was significant in the female group and at a dose <250 mg/day. Moreover, in the intervention with ≥8 weeks and in overweight individuals, the reduction in CRP was significant. OEA improved anthropometric markers, including weight, BMI, fat mass, fat percentage, and waist circumference. The reduction in weight and BMI was significant at a dose ≥250 mg/day and in obese individuals. In addition, the BMI reduction was significant in the intervention ≥8 weeks. OEA improved the levels of oxidative stress markers, including MDA and TAC. The decrease in MDA was significant in the female group and at a dose of <250 mg/day. In the lipid profile, the TG level was significantly reduced, and the reduction in FBG and HOMA-IR was significant. The reduction in FBG was significant at a dose ≥250 mg/day and in obese individuals.

The present meta-analysis showed that the effect of OEA at the dose of <250 mg/day is greater in reducing oxidative and inflammatory stress, while the weight loss and reduction of FBG were significant at the dose of ≥250 mg/day. Therefore, further studies are needed to determine the optimum dose of intervention. Both in the case of reducing inflammation and BMI, long-term intervention for more than 8 weeks has been effective. Because inflammation is greater in overweight and obese people, intervention with OEA while reducing weight in these people has also reduced CRP.

OEA had no significant effect on total cholesterol, LDL, HDL, HbA1c, IL-6, and fat-free mass. One reason for this could be the high heterogeneity, which could not be identified by subgroup analysis for IL-6. For the other outcomes, the number of studies was not sufficient to perform subgroup analysis.

The large effect sizes observed for TAC, MDA, and HOMA-IR can be attributed to various reasons, including the existence of publication bias and sensitivity to studies on these outcomes, small sample sizes, and high heterogeneity.

**Figure 6:**
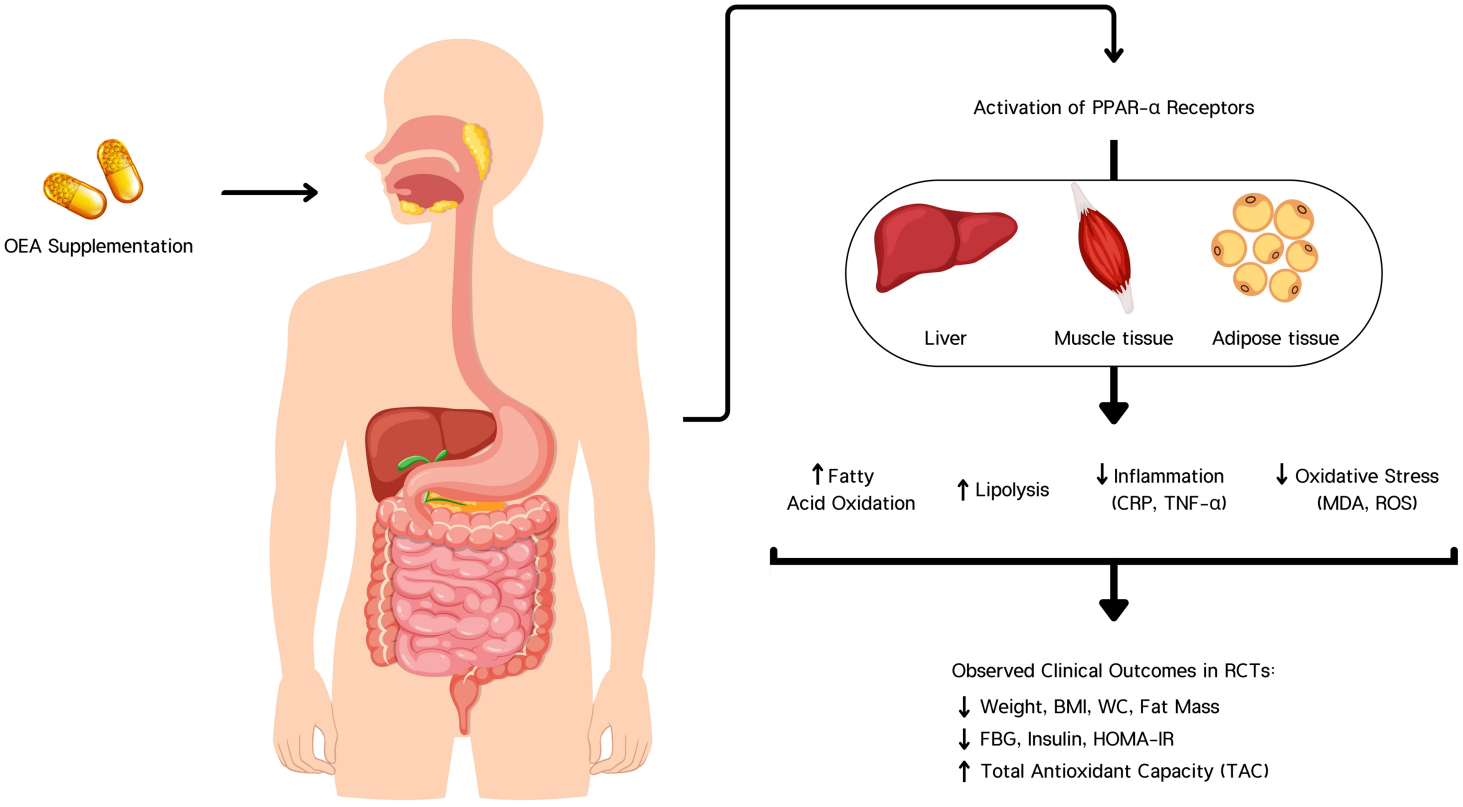
Oleoylethanolamide (OEA) supplementation activates PPAR-α receptors in the liver, muscle, and adipose tissue. This activation enhances fatty acid oxidation and lipolysis, while reducing inflammation (CRP, TNF-α) and oxidative stress (MDA, ROS). These biological effects contribute to clinically observed outcomes in randomized controlled trials (RCTs), including reductions in body weight, BMI, waist circumference (WC), fat mass, fasting blood glucose (FBG), insulin, and HOMA-IR, along with an increase in total antioxidant capacity (TAC).

Two systematic reviews that investigated the effects of OEA on risk factors for non-alcoholic fatty liver disease and obesity management (34, 35) showed that OEA regulates pathophysiological pathways involved in NAFLD, including lipid metabolism, inflammation, oxidative stress, and energy homeostasis (34). OEA is also considered a key component in regulating dietary fat intake and energy homeostasis (Figure 6). Therefore, it could play a role as a therapeutic agent for obesity management (35). The results of these studies are consistent with the present study. However, in the present review, human RCTs were reviewed, and quantitative analysis was performed.

The study by Sabahi et al. was not included in the quantitative analysis due to the short intervention period (3 days). This study was conducted in patients with acute ischemic stroke, and two doses of 600 and 300 mg/day were investigated. The results showed improvement in lipid and inflammatory profiles and oxidative stress with a dose of 300 mg/day (33).

Dietary oleic acid enters the enterocyte via the fatty acid translocase/cluster of differentiation 36 (FAT/CD36) glycoprotein and is converted to N-oleoyl-phosphatidylethanolamine (NOPE) by calcium-dependent N-acetyltransferase (NAT) (36). NOPE is then hydrolyzed by N-acyl-phosphatidylethanolamine phospholipase D (NAPE-PLD) to generate endogenous OEA (37, 38).

OEA activates PPAR-α, leading to the upregulation of genes that enhance fatty acid oxidation and energy expenditure while downregulating genes involved in inflammation (31, 39). OEA inhibits the activation of the NF-κB pathway (40). OEA is structurally similar to anandamide, an endocannabinoid. Unlike anandamide, OEA does not activate cannabinoid receptors (CB1 and CB2) (34, 41). Instead, it exerts its effects through PPAR-α and other pathways, indirectly modulating the endocannabinoid system. By reducing the levels of anandamide, which can promote inflammation through CB1 receptor activation, OEA helps decrease inflammation and associated oxidative stress (34). It enhances the expression of antioxidant enzymes such as superoxide dismutase (SOD), catalase, and glutathione peroxidase through PPAR-α activation (31). It also reduces the production of reactive oxygen species (ROS) by inhibiting enzymes involved in their generation. By enhancing autophagy, OEA helps maintain cellular homeostasis and reduce the accumulation of damaged proteins and organelles, contributing to lower oxidative stress and inflammation (42).

OEA has been shown to stimulate the secretion of GLP-1 from the intestinal L-cells. Increased levels of GLP-1 improve insulin secretion in response to meals, reduce glucagon levels, slow gastric emptying, and enhance satiety (35). Enhanced levels of beneficial adipokines (such as adiponectin) and reduced levels of detrimental adipokines (such as resistin) by OEA improve insulin sensitivity (34). Adiponectin, in particular, enhances glucose uptake and fatty acid oxidation, contributing to lower blood glucose levels and improved insulin sensitivity (43). OEA directly, in muscle, increases fatty acid oxidation, improves insulin sensitivity, and enhances glucose uptake and utilization. In the liver, it reduces lipid accumulation and improves insulin signaling, reducing hepatic glucose production (34).

OEA activates sensory fibers in the vagus nerve, which transmits signals to the brain, specifically to the hypothalamus (35). This activation leads to the release of hedonic and homeostatic hormones and neurotransmitters (dopamine, oxytocin, and histamine) (44). Activation of PPAR-α by OEA increases the expression of genes involved in mitochondrial biogenesis and energy metabolism, enhancing the body’s ability to burn calories (35).

### 4.1 Adverse events

OEA supplementation is typically safe and well-tolerated, with mild gastrointestinal disturbances being the most commonly reported adverse effects. Potential issues such as drug interactions and hypersensitivity reactions should be monitored, especially in individuals with specific health conditions (44). Due to the lack of extensive long-term safety data, it is advisable to use OEA supplements under the guidance of a healthcare professional and to monitor for any adverse effects or changes in health status.

### 4.2 Strengths and limitations

To the best of our knowledge, the present meta-analysis is the first comprehensive quantitative review in which the effect of OEA on cardiometabolic factors has been investigated. The strengths of the study include subgroup analysis, certainty assessment of the evidence according to the GRADE framework, and performing publication bias and sensitivity tests. However, our review included some limitations, such as the limited number of eligible RCTs, two included trials with a high overall risk of bias, and two with some concerns overall risk of bias, high heterogeneity among the pooled effect size for some outcomes, and the limited geographical area where all the studies were conducted in Iran. Geographic differences in diet, genetics, and environment critically shape OEA’s bioavailability and health effects. The limited number of included studies decreases the precision of the pooled effect estimate, leading to wider confidence intervals and a higher likelihood of false-negative results (Type II error). Moreover, sensitivity and publication bias analyses showed that the results obtained were not certain for some of the outcomes studied. Therefore, this meta-analysis may fail to detect an actual treatment effect due to insufficient data.

## 5 Conclusion

Supplementation with OEA may be helpful in improving glycemic control, weight loss, waist circumference, fat mass, fat percentage, inflammation, and oxidative stress. However, given the limitations mentioned in this review, no definitive conclusions can be drawn, and further studies are needed to determine the optimal dose and duration of effective supplementation and to draw more definitive conclusions.

## Data Availability

The original contributions presented in the study are included in the article/Supplementary material, further inquiries can be directed to the corresponding author.
