# Supplementary material for: Oleoylethanolamide supplementation on cardiometabolic health: a systematic review and meta-analysis of randomized controlled trials
Source: Front Nutr. 2025 May 21;12:1553288. doi: 10.3389/fnut.2025.1553288 (PMC12133512; doi:10.3389/fnut.2025.1553288)
Supplement: Supplementary file 1 [file Image_1.pdf]

A)

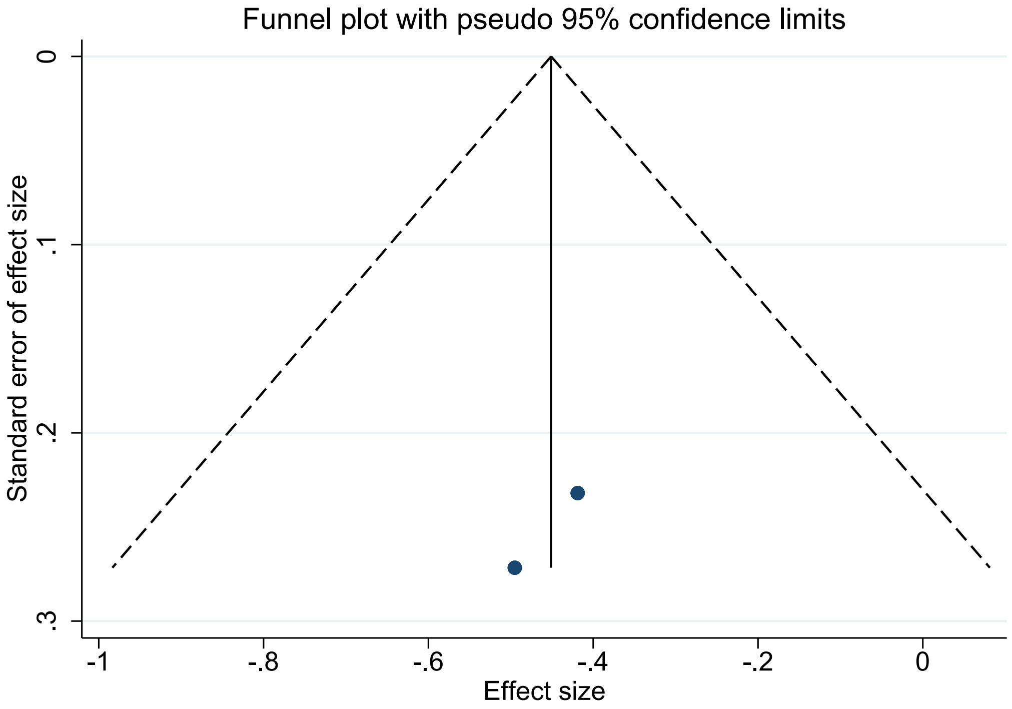
Funnel plot with pseudo 95% confidence limits
Standard error of effect size
0
.1
.2
.3
-1
-.8
-.6
-.4
-.2
0
Effect size

B)

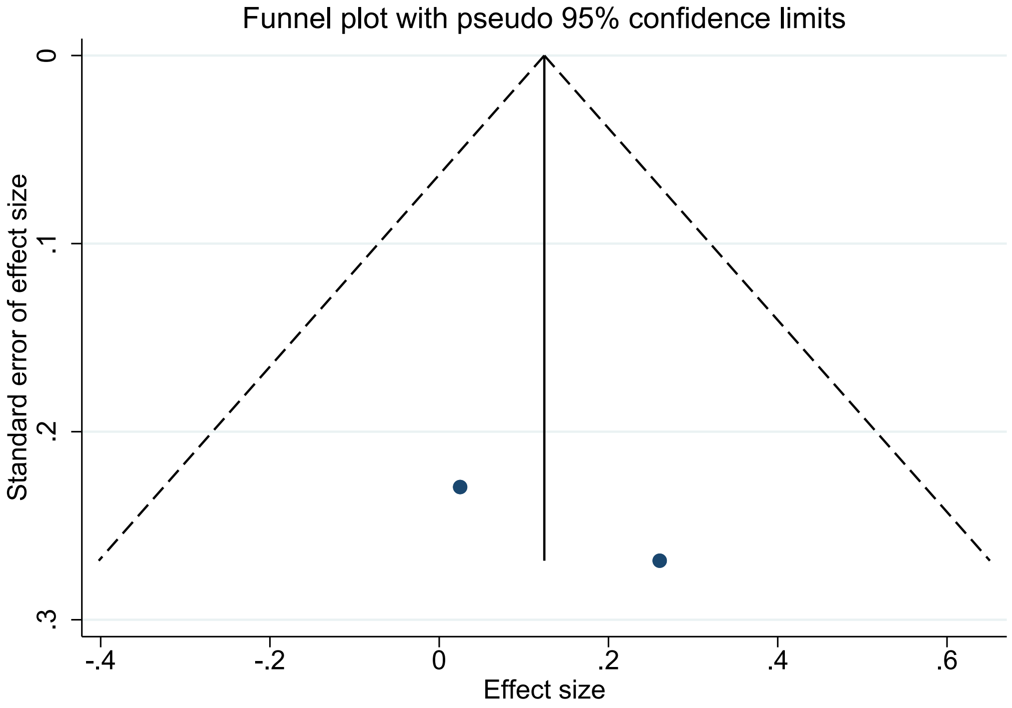
Funnel plot with pseudo 95% confidence limits
Standard error of effect size
0
.1
.2
.3
-.4
-.2
0
.2
.4
.6
Effect size

C)

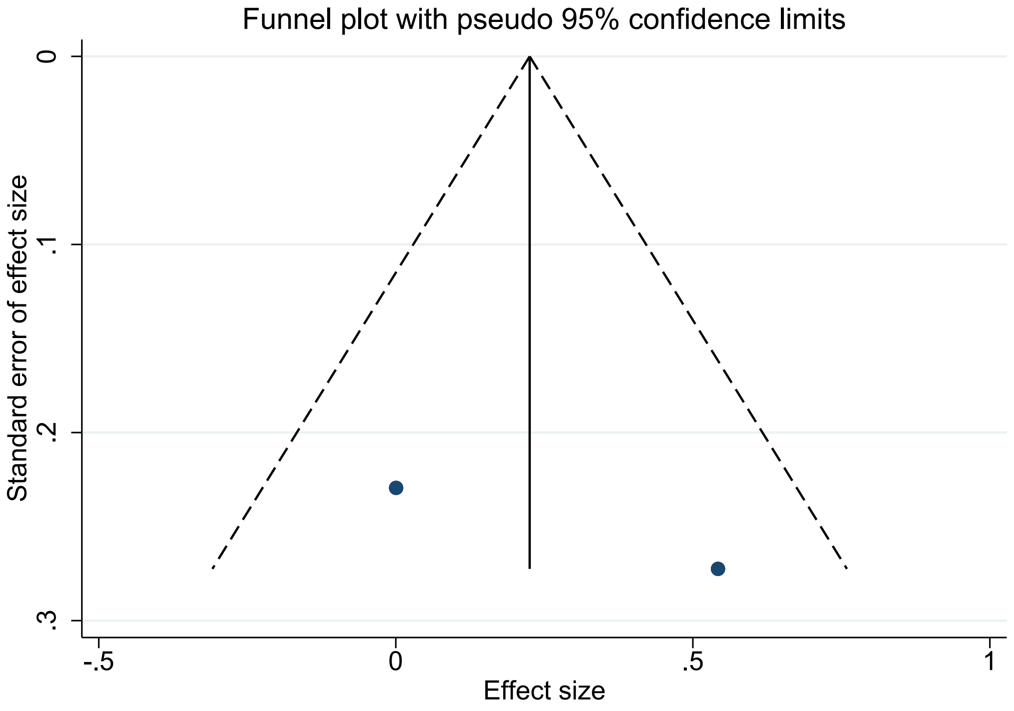

Funnel plot with pseudo 95% confidence limits
Standard error of effect size
0
.1
.2
.3
-.5
0
.5
1
Effect size


D)

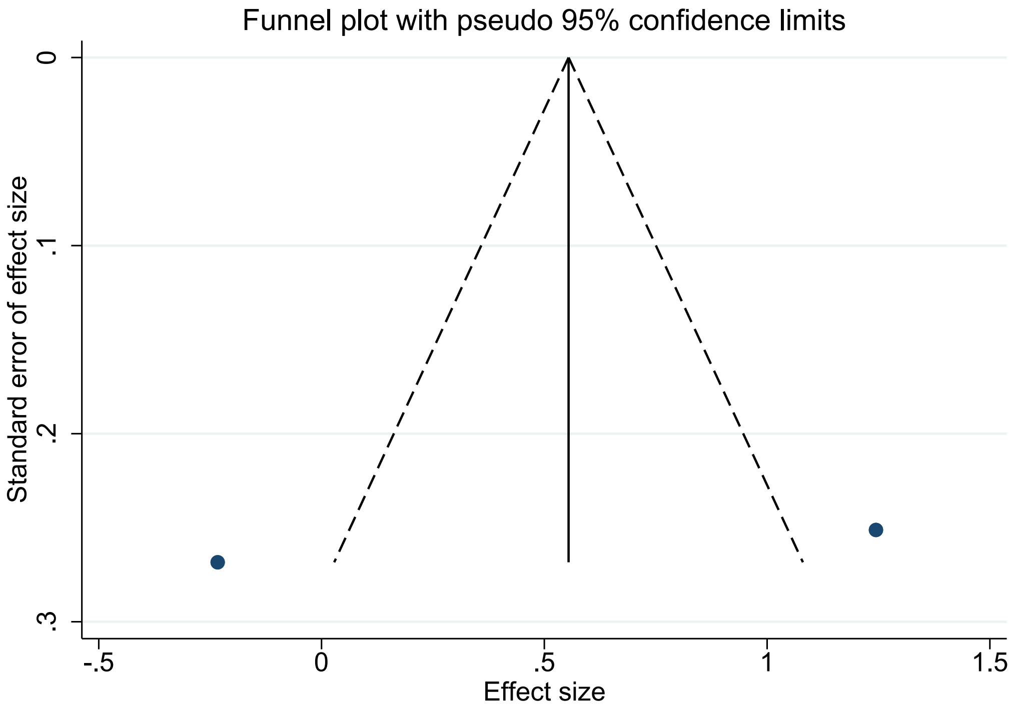

Funnel plot with pseudo 95% confidence limits
Standard error of effect size
0
.1
.2
.3
-.5
0
.5
1
1.5
Effect size

E)

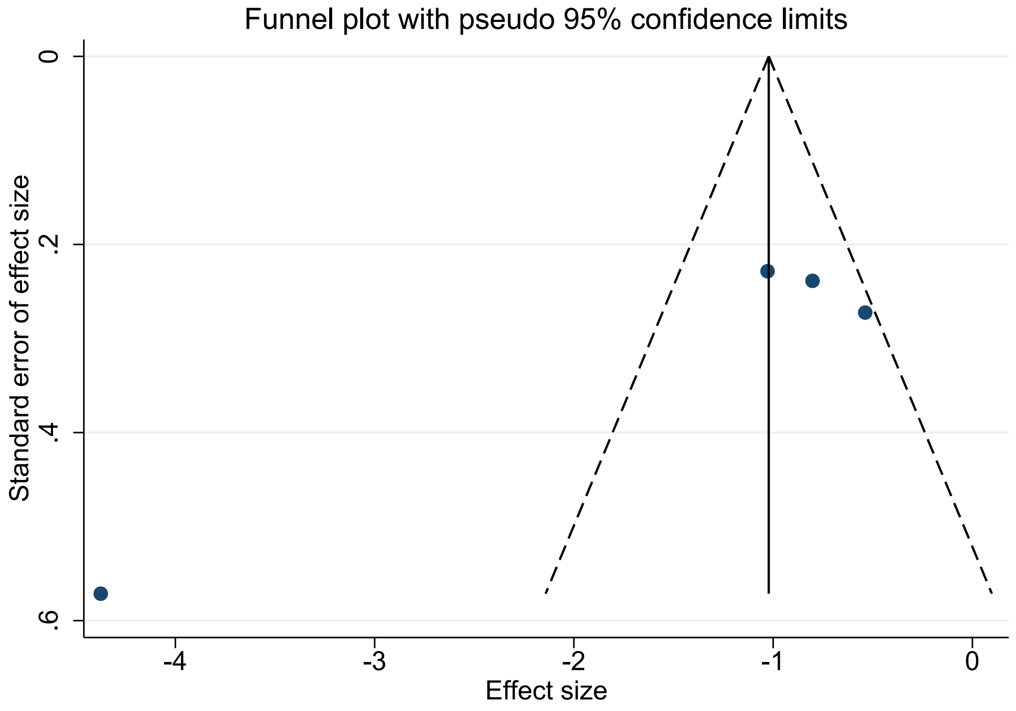

Funnel plot with pseudo 95% confidence limits
Standard error of effect size
0
.2
.4
.6
-4
-3
-2
-1
0
Effect size


F)

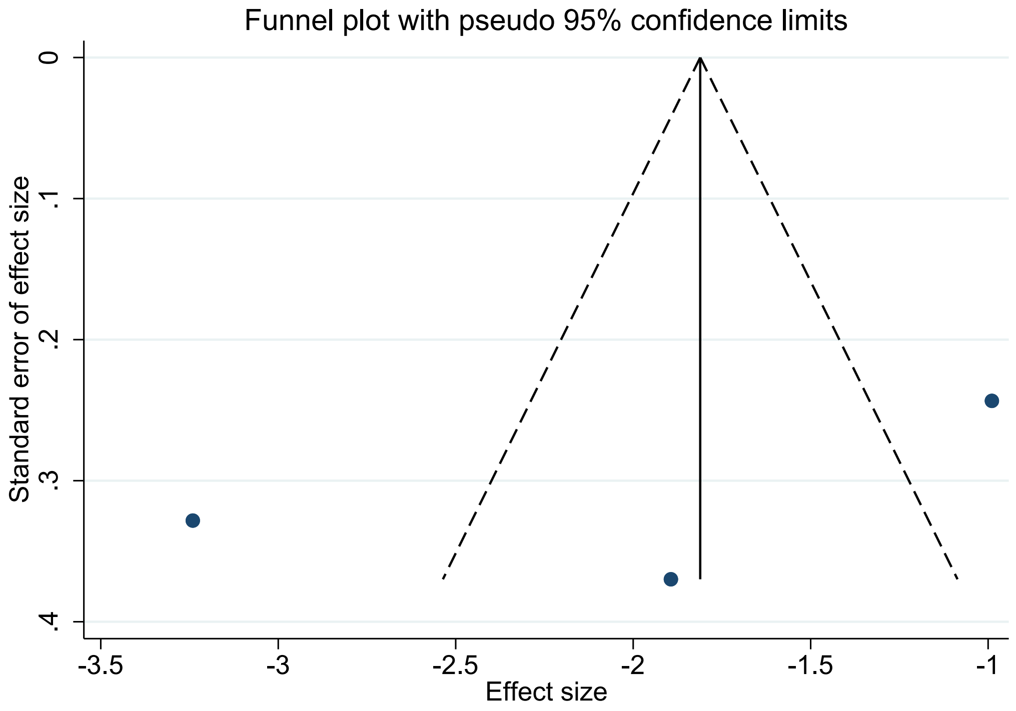

Funnel plot with pseudo 95% confidence limits
Standard error of effect size
0
.1
.2
.3
.4
-3.5
-3
-2.5
-2
-1.5
-1
Effect size

G)

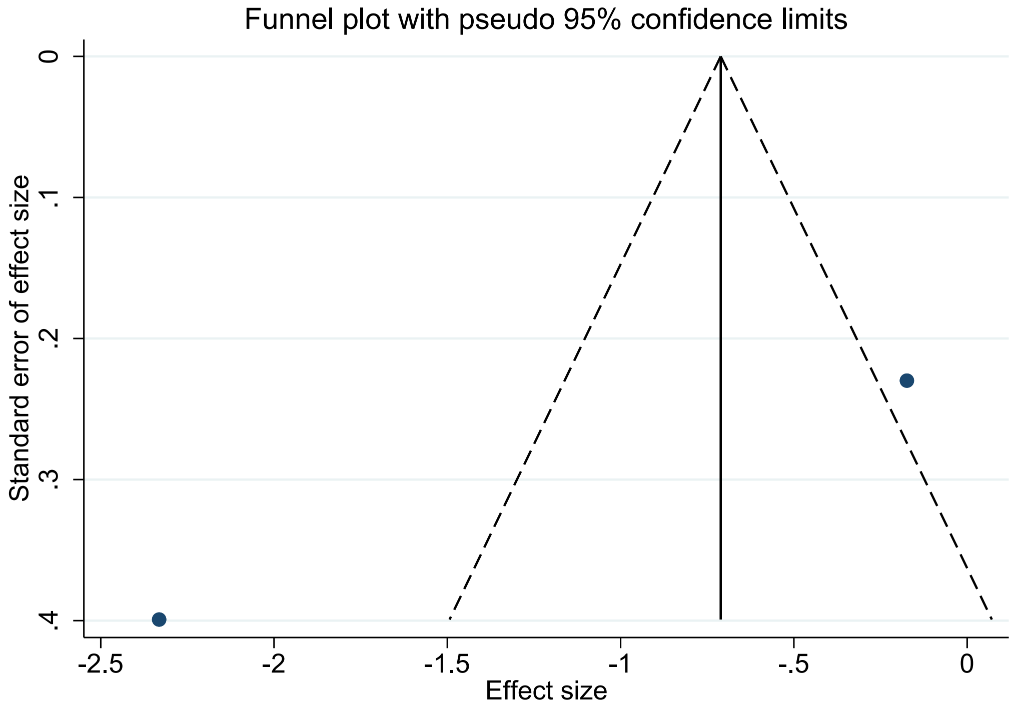
Funnel plot with pseudo 95% confidence limits
Standard error of effect size
0
.1
.2
.3
.4
-2.5
-2
-1.5
-1
-.5
0
Effect size

H)

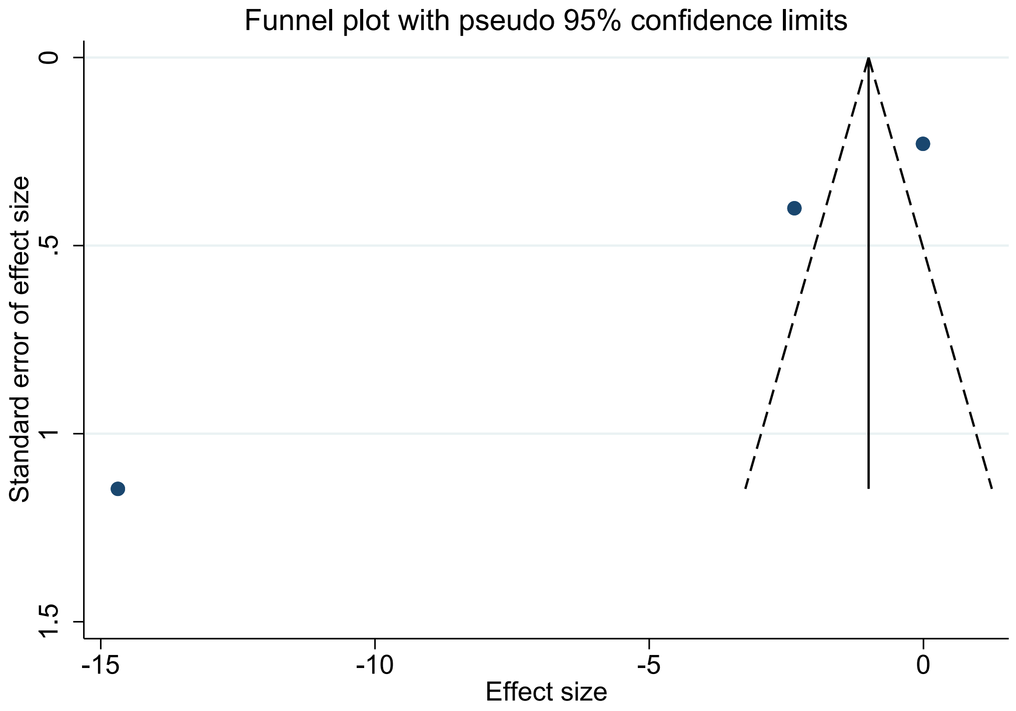
Funnel plot with pseudo 95% confidence limits
Standard error of effect size
0
.5
1
1.5
-15
-10
-5
0
Effect size

I)

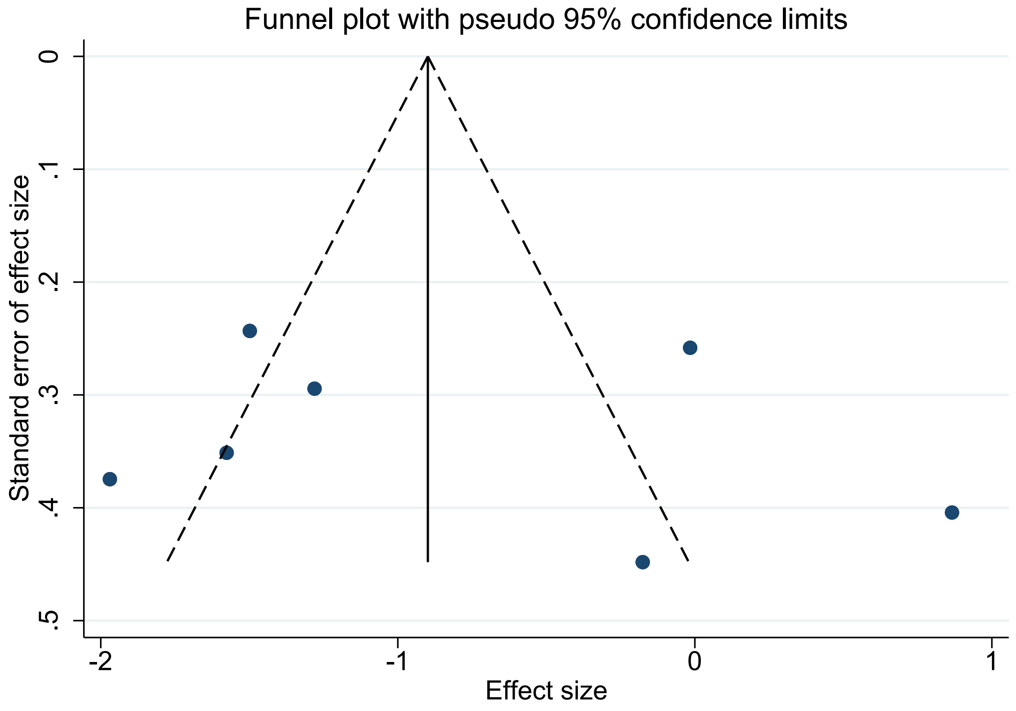
Funnel plot with pseudo 95% confidence limits
Standard error of effect size
0
.1
.2
.3
.4
.5
-2
-1
0
1
Effect size

J)

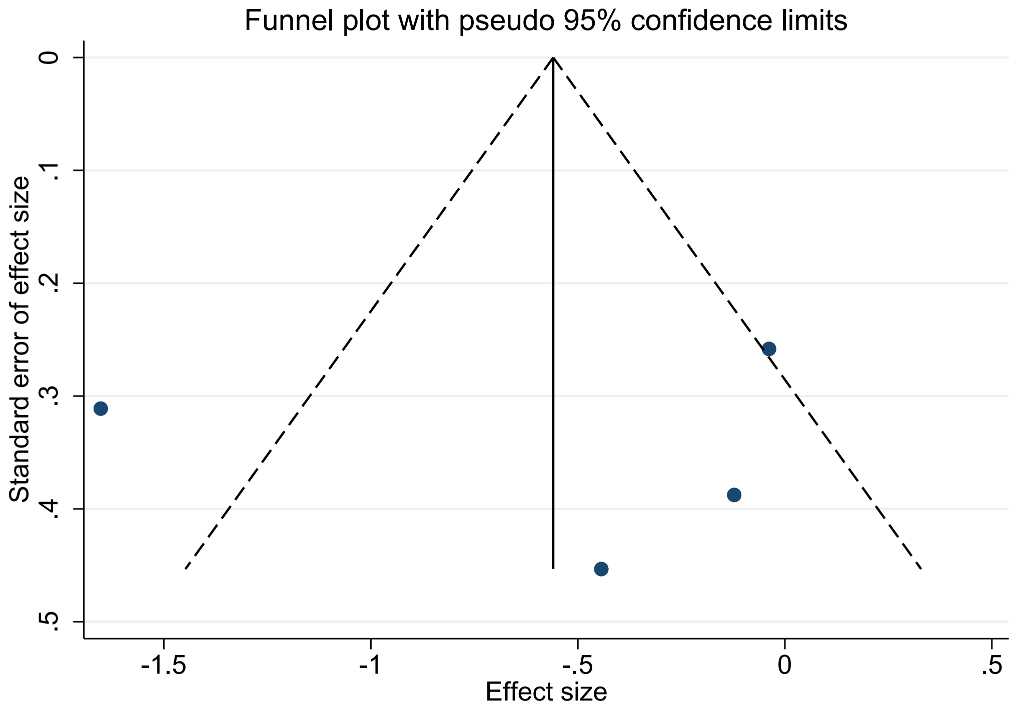
Funnel plot with pseudo 95% confidence limits
Standard error of effect size
0
.1
.2
.3
.4
.5
-1.5
-1
-.5
0
.5
Effect size

K)

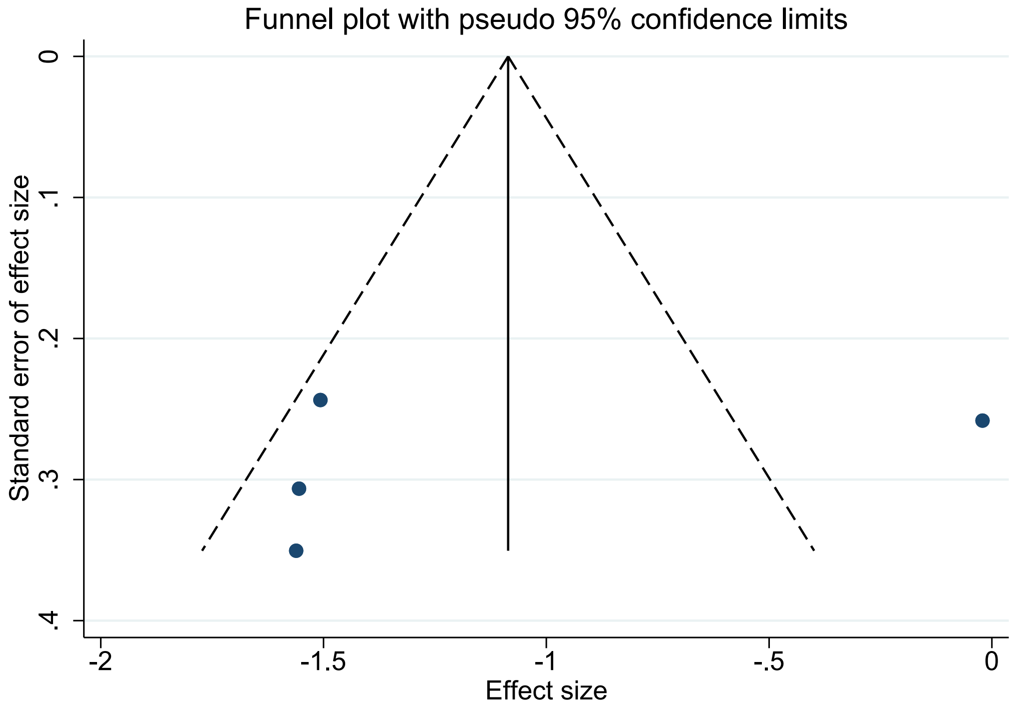

Funnel plot with pseudo 95% confidence limits
Standard error of effect size
0
.1
.2
.3
.4
-2
-1.5
-1
-.5
0
Effect size


L)

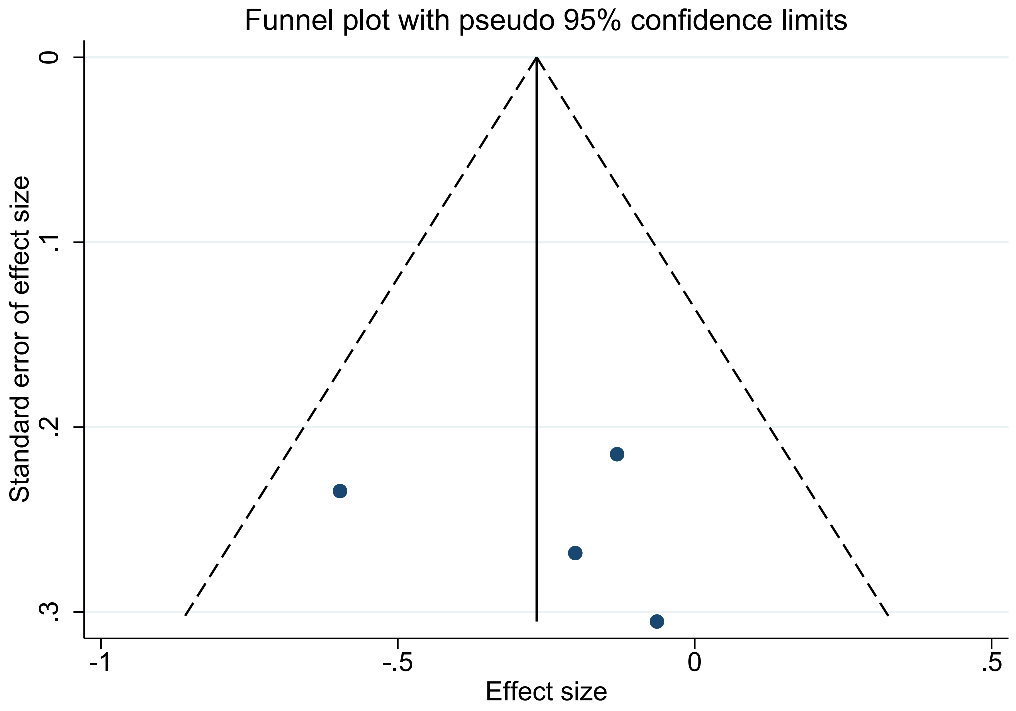

Funnel plot with pseudo 95% confidence limits
Standard error of effect size
0
.1
.2
.3
-1
-.5
0
.5
Effect size

M)

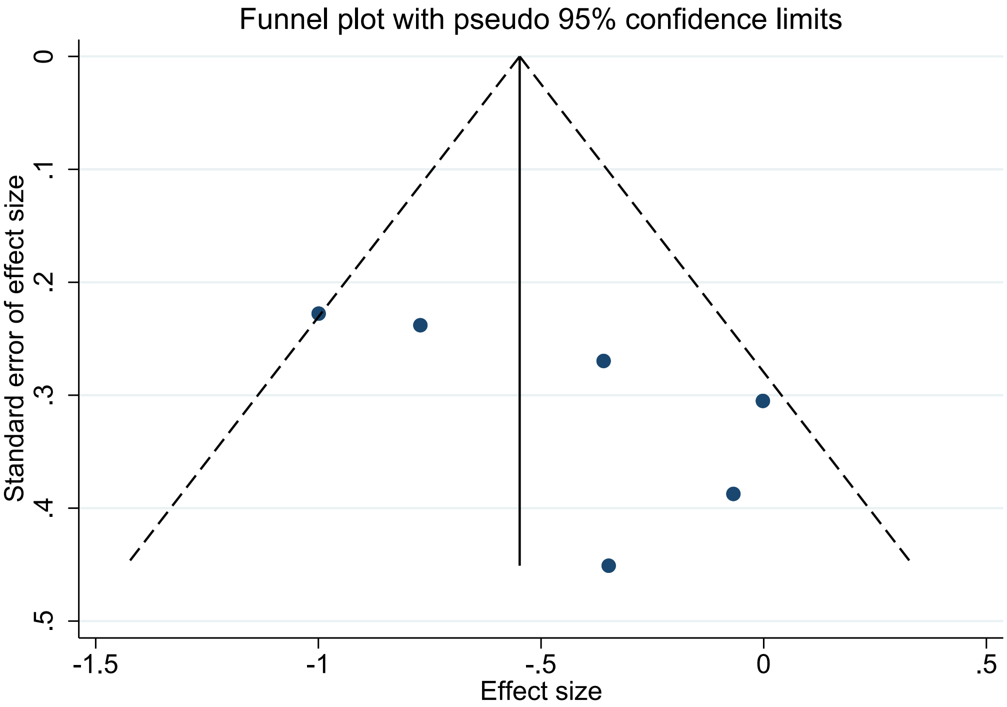

Funnel plot with pseudo 95% confidence limits
Standard error of effect size
0
.1
.2
.3
.4
.5
-1.5
-1
-.5
0
.5
Effect size


N)

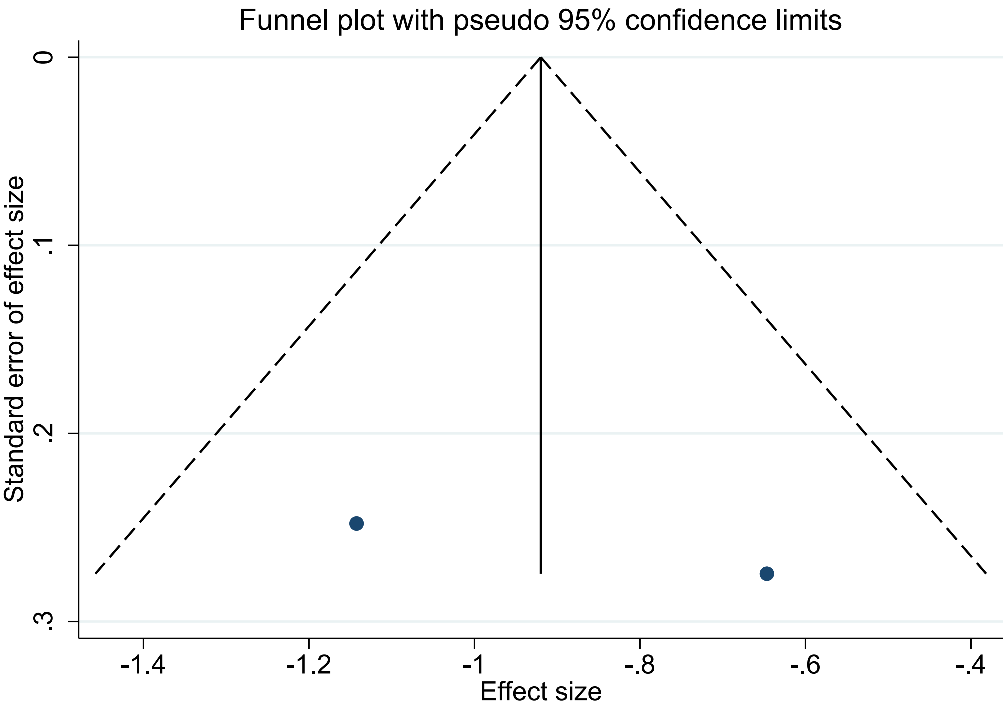

Funnel plot with pseudo 95% confidence limits
Standard error of effect size
0
.1
.2
.3
-1.4
-1.2
-1
-.8
-.6
-.4
Effect size

**O)**

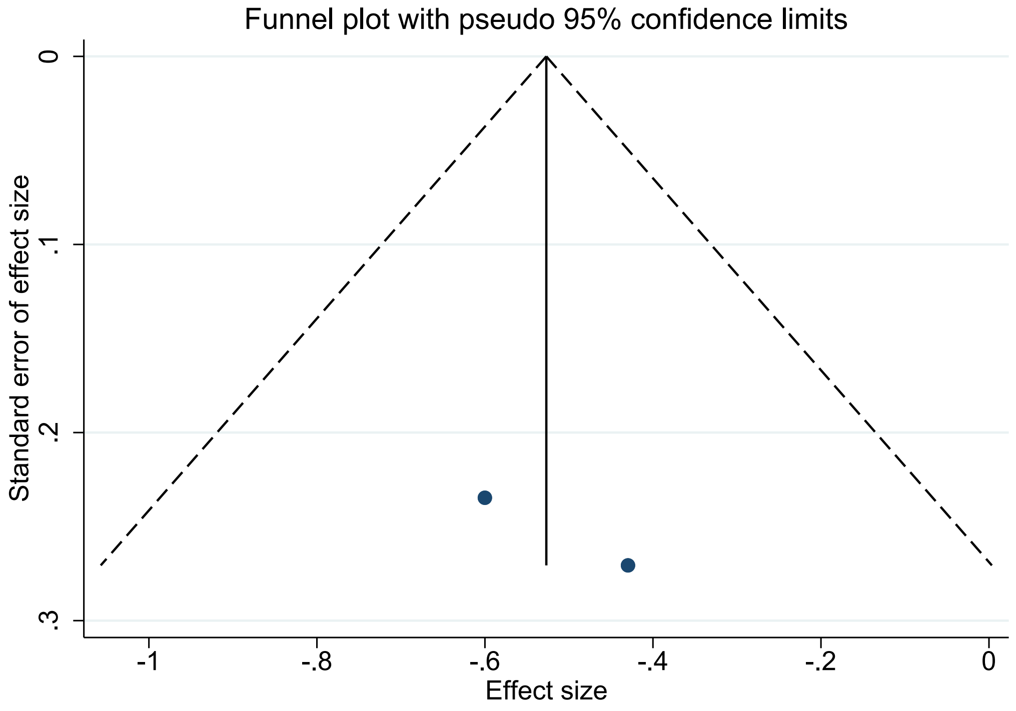

Funnel plot with pseudo 95% confidence limits
Standard error of effect size
0
.1
.2
.3
-1
-.8
-.6
-.4
-.2
0
Effect size


**P)**

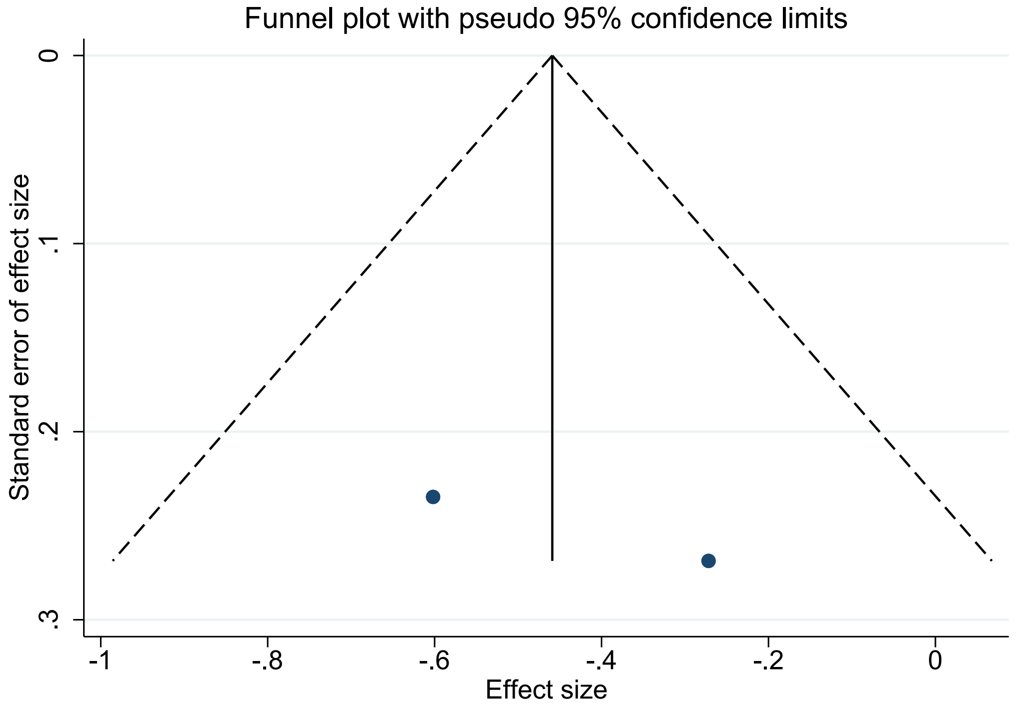

Funnel plot with pseudo 95% confidence limits
Standard error of effect size
0
.1
.2
.3
-1
-.8
-.6
-.4
-.2
0
Effect size

**Q)**

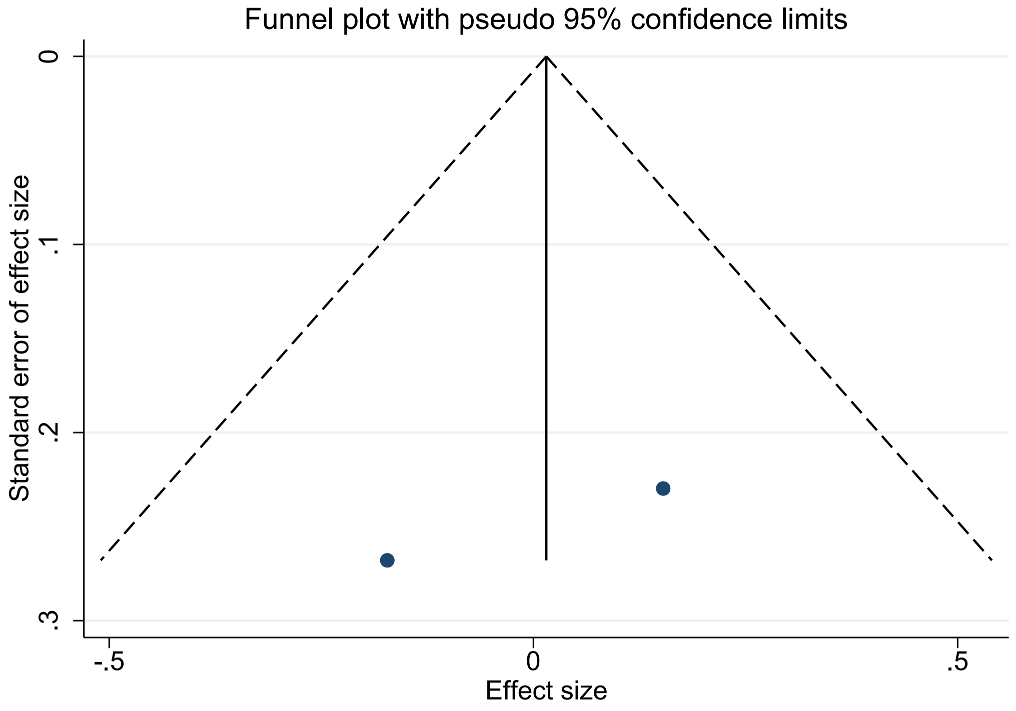
Funnel plot with pseudo 95% confidence limits
Standard error of effect size
0
.1
.2
.3
-.5
0
.5
Effect size

**R)**

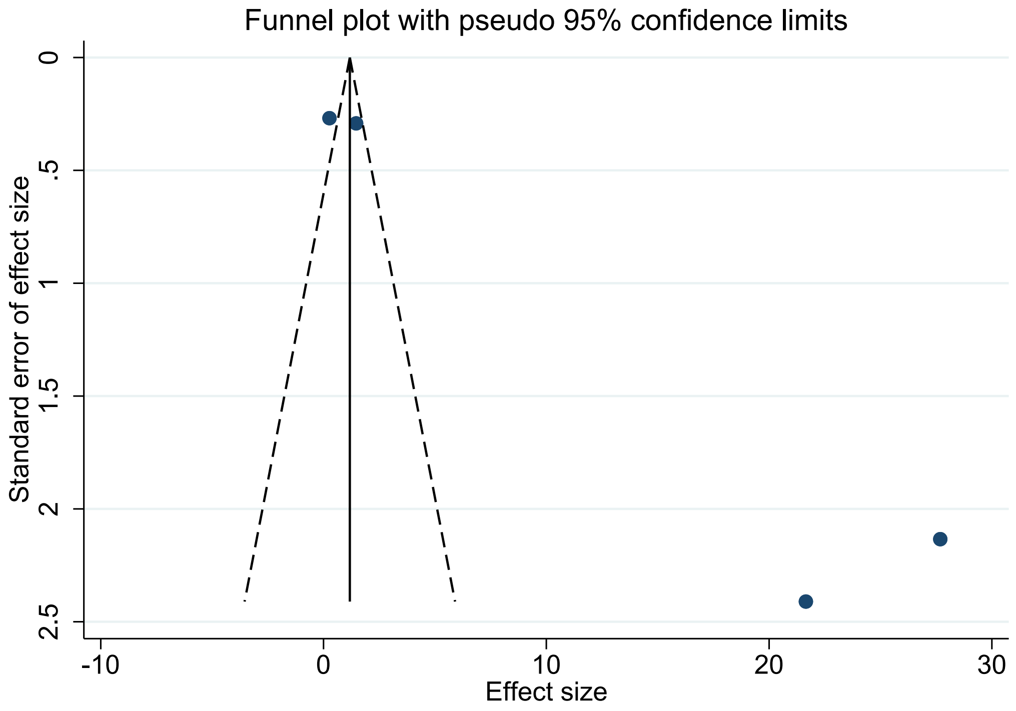
Funnel plot with pseudo 95% confidence limits
Standard error of effect size
0
.5
1
1.5
2
2.5
-10
0
10
20
30
Effect size

**S)**

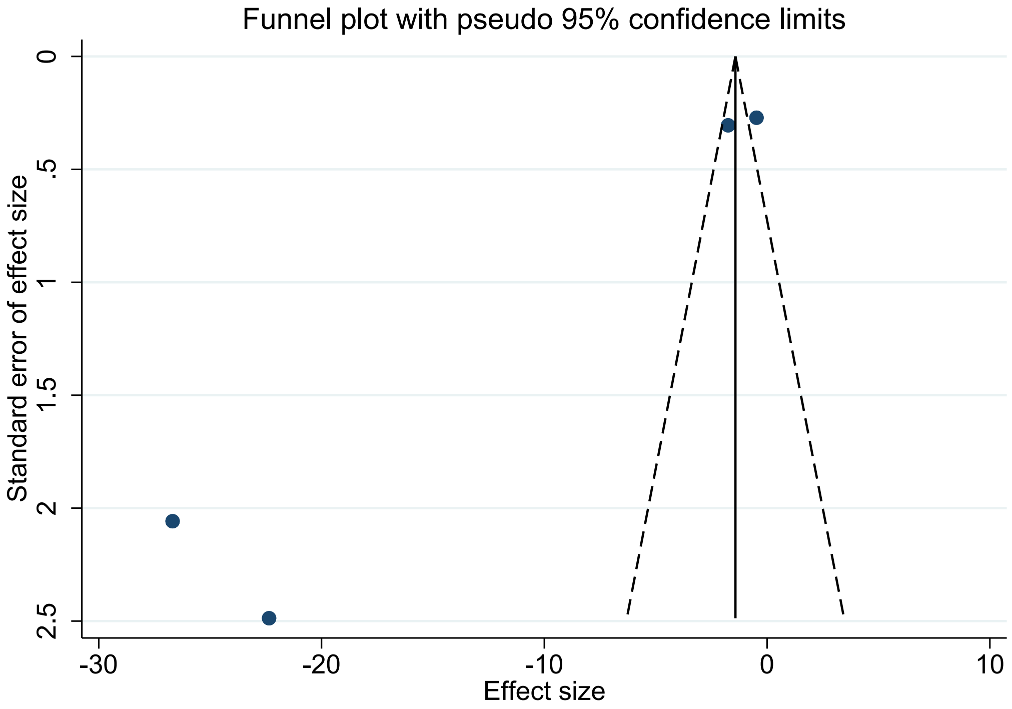


**Supplementary Figure 1.** Funnel plots for the effect of oleoylethanolamide intake on A) triglycerides; B) total cholesterol; C) LDL-C ; D) HDL-C; E) FBG; F) Insulin; G) HbA1c; H) HOMA-IR; I) CRP; J) IL-6; K) TNF-α; L) body weight; M) BMI; N) waist circumference; O) fat mass; P) body fat percentage; Q) fat-free mass; R) TAC; S) MDA.
